# A multicellular vessel-on-a-chip model reveals context-dependent roles for platelets in inflammation and inflammatory hemostasis

**DOI:** 10.1016/j.bvth.2024.100007

**Published:** 2024-04-30

**Authors:** Rebecca B. Riddle, Karin Jennbacken, Kenny M. Hansson, Matthew T. Harper

**Affiliations:** 1 Department of Pharmacology, University of Cambridge, Cambridge, United Kingdom; 2 Research and Early Development, Cardiovascular, Renal and Metabolism, R&D BioPharmaceuticals, AstraZeneca, Gothenburg, Sweden

## Abstract

The immune system identifies and destroys invading pathogens. However, chronic inflammation and excessive tissue damage can occur if inflammation is not resolved or if the immune system misidentifies a threat. Therefore, the immune system must be tightly controlled. However, the complex cell-cell and cell-microenvironment interactions that regulate immune-cell recruitment are challenging to recapitulate in 2-dimensional in vitro models, and poorly representative of human physiology in in vivo animal models. Organ-on-a-chip technology has the potential to overcome these limitations and provide powerful preclinical models. In this study, we developed a vessel-on-a-chip model to investigate the role of platelets in inflammation and inflammatory hemostasis. Endothelial vessels cultured in the OrganoPlate exhibited physiological barrier function to macromolecules and red blood cells (RBCs), which was enhanced by platelets. Cytokine stimulation increased vessel permeability and induced neutrophil transmigration. Leakage of RBCs occurred at sites of inflammation and/or neutrophil transmigration, depending on the extracellular matrix composition. Addition of platelets prevented RBC leakage at these sites, while simultaneously increasing permeability and neutrophil transmigration, demonstrating the multifaceted role of platelets in inflammatory hemostasis. In angiogenic neovessels, platelets played a protective role, preventing leakage of both small molecules and RBCs. Together, our model successfully recapitulated the modulation of endothelial barrier function and inflammation by platelets and demonstrated the feasibility of investigating complex cellular interactions in in vitro models.

## Key Point

• Our vessel-on-a-chip model recapitulates the multifaceted roles of platelets in neutrophil transmigration and inflammatory hemostasis.

## Visual Abstract

**Figure:**
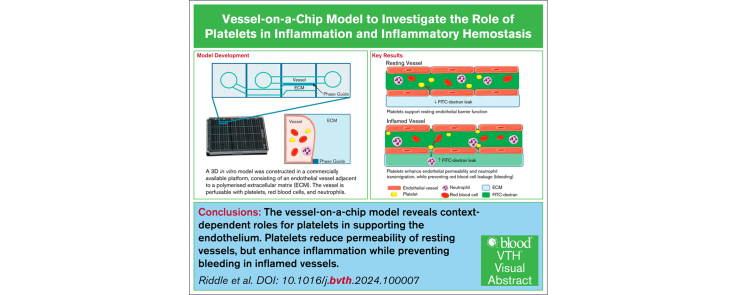

## Introduction

The immune system identifies and destroys invading pathogens. Circulating immune cells such as neutrophils are recruited from the blood, cross the vascular endothelium, and migrate through the extracellular matrix (ECM) toward the source of inflammation. There they eliminate pathogens through secretion of cytotoxic granules, production of reactive oxygen species, and release of extracellular DNA.^1^^,^^2^ However, chronic inflammation and excessive tissue damage can result if inflammation is not resolved or if the immune system misidentifies a threat.^3^ There are many pathological conditions characterized by excessive inflammation, including coronary artery disease, multiple sclerosis, and rheumatoid arthritis. In other conditions, inflammation plays a major role in disease progression, including cancer and severe COVID-19.^4^ To treat these diseases, the mechanisms by which immune cells are recruited, cross the vascular endothelium, and induce tissue damage must be understood.

Until recently, research into inflammation heavily relied upon in vivo animal models or simple in vitro models. Animal models allow the study of the complex multicellular interactions that occur during inflammation but there are critical differences between the innate immune system of mice and humans that limit the translational impact of these models. For example, neutrophils make up ∼50% to 70% of human leukocytes but only 10% to 25% of murine leukocytes.^5^ The expression of key proteins that contribute to inflammation such as Toll-like receptors, chemokine receptors, and myeloperoxidase, also varies significantly between humans and mice.^6^ Classic in vitro models typically consist of 2-dimensional (2D) monolayers of human cell lines cultured on tissue culture–treated plastic.^7^ However, these models do not accurately recapitulate in vivo microenvironments. Recent developments in bioengineering have led to the creation of “organ-on-a-chip” models, a technology whereby cells can be grown in microchip-sized devices that recapitulate physiological 3D microenvironments.^8^ Organ-on-a-chip models have the potential to become a powerful tool in basic science and drug discovery.^9^^,^^10^ For example, we previously reported development of an inflammation-on-a-chip model of neutrophil transmigration and subsequent ECM migration,^11^ and demonstrated that endothelial inflammation and neutrophil infiltration are modulated by ECM composition. One of the strengths of organ-on-a-chip models is that individual components can be precisely controlled to investigate their role.

Platelets maintain hemostasis by aggregating at sites of vessel injury yet also have an underappreciated role as actors in the immune system. Many studies have highlighted the proinflammatory activities of platelets, including release of cytokines,^12^ recruitment of immune cells,^13^^,^^14^ and induction of endothelial permeability.^15^ Conversely, in unstimulated vessels, platelets appear to have a protective role, reducing endothelial permeability.^16^ Despite these important in vivo roles, the multifaceted contribution of platelets to inflammation is rarely incorporated into in vitro models. Moreover, a key role for platelets in maintaining hemostasis during inflammation has been revealed by mouse models.^16^, ^17^, ^18^, ^19^, ^20^, ^21^, ^22^, ^23^, ^24^ The endothelium normally limits the leakage of large macromolecules and cells from the blood into tissues.^25^ During inflammation, endothelial permeability to macromolecules is increased but red blood cells (RBCs) are retained inside the vessel lumen. However, bleeding is seen at sites of inflammation when platelet count is very low (thrombocytopenia).^17^^,^^22^^,^^26^ Therefore, although platelets promote leukocyte transmigration and endothelial permeability during inflammation, they may additionally prevent leakage of RBCs (bleeding) at these sites. This is a distinct process from conventional hemostasis, which occurs after vessel injury.^19^ This hypothesis has been supported by case studies of patients with thrombocytopenia who exhibited bleeding after inflammatory insults such as sunburn or infection^27^^,^^28^ despite the lack of overt vessel injury. Platelets play a similar role in preventing hemorrhage during angiogenesis.^29^^,^^30^ Therefore, platelets are a key player in inflammation, neutrophil infiltration, and vascular integrity. Platelet interactions with the endothelium, neutrophils, and the ECM may be valuable drug targets and need to be represented in organ-on-a-chip models of inflammation.

Here, we develop a multicellular model of inflammation and inflammatory hemostasis that incorporates platelets and RBCs. This model recapitulates the multifaceted roles of platelets in inflammation and vascular integrity. We also demonstrate that our approach can be used to study the role of platelets in maintaining vascular integrity and hemostasis in angiogenesis.

## Materials and methods

All materials were obtained from Sigma (Poole, United Kingdom) unless specified otherwise.

### ECM compositions

For collagen I ECM, 5 mg/mL rat tail collagen type I (ibidi) was neutralized by mixing 8:1:1 with 1 M HEPES (*N*-2-hydroxyethylpiperazine-*N′*-2-ethanesulfonic acid) and 37 g/L NaHCO_3_ producing a 4-mg/mL solution. For “Geltrex mix” ECM, Geltrex LDEV-Free Reduced Growth Factor Basement Membrane Matrix (Gibco, A1413302) was mixed at a ratio of 75:25 (volume to volume [v/v]) with 3 mg/mL collagen I. Collagen I (3 mg/mL) was made by neutralization of 5 mg/mL collagen I with 1 M NaOH and 7.5 % (weight per volume) NaHCO_3_. Geltrex is a basement membrane extract of murine Engelbreth-Holm-Swarm sarcomas (similar to Matrigel) primarily consisting of laminin, collagen IV, and heparin-sulfate proteoglycans. The precise composition of Geltrex is proprietary.

### OrganoPlate culture

**Figure 1.**
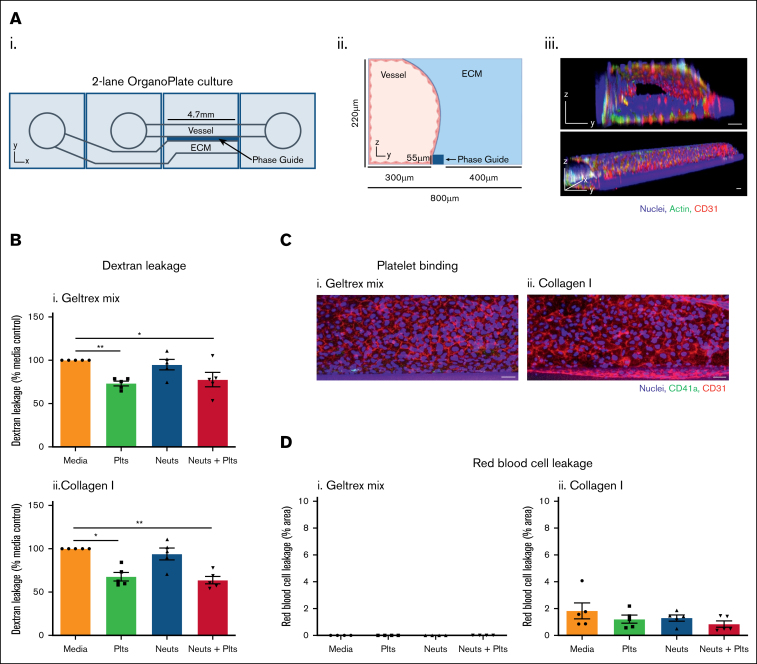
**Platelets reduce leakage of small molecules from 3D HUVEC vessels.** (A) Vessel formation in the 2-lane OrganoPlate. (i) The 2-lane OrganoPlate consists of 96 chips in a 384-well microplate format. Each chip has 2 lanes separated by a phase guide which supports patterning of ECM and cells via inlets and outlets. (ii) Addition of ECM into the bottom channel forms a barrier against which cells can be seeded in the top channel. (iii) HUVEC were seeded into the top channel and cultured for 1 week. Vessels were fixed and then stained with Hoechst-33342 (blue), α-CD31 (red), and phalloidin-FITC to identify actin (green). Images were taken on a Leica SP5 confocal microscope. 3D reconstructions of Z-stacks were produced in FIJI. The PhaseGuide is autofluorescent at 405 nm, and therefore is visible as a blue cuboid structure adjacent to the vessel. (B) Platelets reduce permeability of unstimulated vessels. Vessels were cultured as described for panel A, against Geltrex mix (i) or collagen I (ii). Platelets (plts) and neutrophils (neuts) were isolated from human blood and resuspended in EGM-FCS. They were then perfused through the vessels for 1.5 hours. FITC-dextran (250 kDa) was added for the last 45 minutes and then fluorescence was imaged on an EVOS M5000. Dextran leakage was calculated by dividing the fluorescence in the ECM channel by the fluorescence in the vessel channel. Data were then normalized to the media only control. (C) Platelet adherence is not observed in unstimulated vessels. After the process described in panel B, vessels were fixed, blocked, and stained with Hoechst-33342 (blue), α-CD41a (green), and α-CD31 (red). Z-stacks were taken on a Leica SP5 confocal microscope. (D) Collagen I vessels are leakier to RBCs than Geltrex mix vessels. After neutrophil and platelet perfusion, RBCs were added to the vessels for 30 minutes. RBC leakage was imaged using an iPhone through a bright-field microscope and quantified in FIJI. N = 5 independent experiments per blood donor (N = 4 in panel Ci), n = 2 chips per condition. Mean ± standard error of the mean (SEM); ∗*P* < .05 and ∗∗*P* < .01. Scale bars are representative of 50 μm.

Two-lane OrganoPlates (Mimetas) were used. Human umbilical vein endothelial cell (HUVEC) vessels were cultured as previously described.^11^ Briefly, 1.85 μL ECM was added to the ECM channel via the ECM inlet (Figure 1A). ECMs were polymerized for 10 minutes (37°C, 5% CO_2_), then 50 μL Hanks balanced salt solution was added to the gel inlet to prevent ECM dehydration. Chips were incubated for a further 50 minutes, after which Hanks balanced salt solution was aspirated from the gel inlets.

HUVECs were seeded into the top channel by addition of 2 μL cell suspension at 2 × 10^7^ cells per mL. Media was added to the top channel inlet and the plate incubated for 2 hours on its side at a 70° angle (37°C, 5% CO_2_) to allow cells to attach to the ECM. Media was then added to the top channel outlet and perfusion initiated by interval rocking (8-minute intervals, 7° angle) on the Mimetas OrganoFlow S plate rocker to generate flow by passive liquid levelling. These settings produce a shear stress of up to 2.2 dynes/cm^2^, according to the manufacturer. Plates were incubated under perfusion for 7 days to enable optimal barrier function of the vessels. Media was refreshed every 2 to 3 days.

### Platelet, neutrophil, and RBC isolation

Blood was taken from volunteers by venipuncture into 3.8% (v/v) sodium citrate–containing vacutainers (Greiner Bio-One). The volunteers were healthy and free of medication for at least 10 days before venepuncture. Fully informed, written consent was obtained in accordance with the Declaration of Helsinki, and use of blood was reviewed by the Human Biology Research Ethics Committee, University of Cambridge. Washed platelets were prepared by centrifugation as previously described.^31^ Neutrophils were isolated by centrifugation on Histopaque-1077 and dextran sedimentation as previously reported.^11^ RBCs were taken from the neutrophil preparation, after sedimentation in 2% (v/v) dextran.

### Barrier-integrity assay

Fluorescein isothiocyanate (FITC)-dextran (0.5 mg/mL, 250 kDa) was added to the top channel for 45 minutes. Dye retention was imaged on an EVOS M5000 microscope. Barrier function was quantified by measuring the fluorescence in each channel in Fiji^32^ and then calculating the ratio of fluorescence in the vessel vs fluorescence in the ECM (supplemental Figure 2A,C).

### Neutrophil-platelet-RBC cocultures

On day 7 of culture, vessel lumens were stimulated overnight with 10 ng/mL human recombinant tumor necrosis factor α (TNF-α; R&D Systems) and 10 ng/mL human recombinant interleukin 1β (IL-1β; R&D Systems). Neutrophils and/or platelets resuspended in endothelial cell growth medium (EGM; PromoCell) + 10% (v/v) fetal calf serum (FCS) were then added to the vessels for 1.5 hours on the rocker (37 °C, 5% CO_2_). The final cell concentrations were 2 × 10^8^ platelets per mL and 4 × 10^6^ neutrophils per mL. FITC-dextran was added to the vessels for the last 45 minutes for the barrier integrity assay. RBCs were then added to the vessels for 30 minutes before imaging RBC leakage (supplemental Figure 2B) using an iPhone attached to a bright-field microscope. RBCs were used at 10% hematocrit.

### Angiogenesis

Three-lane OrganoPlates (Mimetas) were used. Vessels were plated as described previously,^11^ and cultured for 2 days in EGM supplemented with 30 ng/mL vascular endothelial growth factor (PeproTech) and 20 ng/mL basic fibroblast growth factor (PeproTech), before adding an angiogenesis-inducing cocktail into the bottom channel. The cocktail consisted of EGM containing 50 ng/mL vascular endothelial growth factor, 500 nM sphingosine 1-phosphate (S1P), 2 ng/mL phorbol 12-myristate 13-acetate (PMA), and 22 ng/mL basic fibroblast growth factor. Media and angiogenesis factors were refreshed every 2 to 3 days. Platelets were added 8 days after induction of angiogenesis. Vessels were stained with CellTracker Deep Red Plasma Membrane stain (ThermoFisher) before platelet addition. Bleeding was quantified based on presence of extravascular CD235a^+^ RBCs not colocalized with the CellTracker Deep Red Plasma Membrane stain.

### Immunofluorescence

Immunofluorescence staining and neutrophil transmigration quantification was performed as previously reported.^11^

### Statistical analysis

Statistical analysis was performed in GraphPad Prism version 9. All tests were carried out using matched analyses. Two-way analysis of variance with Šidák multiple comparisons test was used to analyze differences in dextran leakage, RBC leakage, and neutrophil transmigration with neutrophils and/or platelets. Paired 2-tailed *t* tests were used to analyze differences in dextran leakage between stimulated and unstimulated, or to analyze differences in dextran and RBC leakage with platelets in angiogenesis experiments. *P* <.05 was considered significant. In figure legends, “N” refers to independent experiments/blood donors, and “n” refers to technical repeats (number of vessels) within donors.

## Results

### Platelets prevent leakage of macromolecules from unstimulated 3D endothelial vessels

To investigate how circulating blood cells affect endothelial vessel integrity, we developed a 3D inflammation-on-a-chip model. The model was constructed in the 2-lane OrganoPlate (Mimetas; Figure 1Ai). These devices contain 2 channels separated by a “phase guide,” a raised ridge that separates the vessel and ECM channels. The phase guide pins liquid ECM, preventing it spilling into the vessel channel. After ECM polymerization there is a solid barrier of ECM above the phase guide, against which cells can be directly seeded. In our model, HUVECs were seeded in the vessel channel against either collagen I or Geltrex mix (75% Geltrex + 25% collagen I) ECMs for 1 week (Figure 1Aii). The HUVECs formed confluent vessels, which grew on all 4 sides of the channel (Figure 1Aiii). Platelets and neutrophils were isolated from healthy human donors and resuspended in endothelial growth medium supplemented with FCS (EGM-FCS), a media in which platelets, neutrophils, and RBCs retained viability (supplemental Figure 1). Unstimulated vessels were perfused with platelets and/or neutrophils, or EGM-FCS as control, for 90 minutes. Therefore, in our model, the stable endothelial vessel was allowed to form in the absence of platelets or neutrophils, and so any effect of these blood cells represents an effect on the mature vessel. This allows us to exclude effects on HUVEC proliferation.

To assess vessel permeability to macromolecules, the vessel channel was perfused with FITC-dextran (250 kDa). Although most fluorescence was retained in the vessel channel, some was detected in the ECM channel, indicating FITC-dextran leakage. Platelet perfusion significantly reduced leakage of FITC-dextran from both Geltrex mix and collagen I vessels (Figure 1B; supplemental Figures 3A and 4A). This demonstrates that platelets protect blood vessel integrity, preventing macromolecule leakage from unstimulated vessels. In contrast, neutrophils had no effect (Figure 1B). Immunofluorescence imaging revealed very little platelet adhesion to the HUVECs (Figure 1C), suggesting this effect was not due to physically blocking leakage from the vessels.

When RBCs were perfused through the vessels, no leakage was observed from Geltrex mix vessels (Figure 1Di; supplemental Figure 3A). In contrast, a low level of RBC leakage occurred from unstimulated collagen I vessels (Figure 1Dii; supplemental Figure 4A). This suggests that the ECM composition can modulate the resting vessel barrier function to larger cells.

### Platelets promote inflammation while preventing RBC leakage

**Figure 2.**
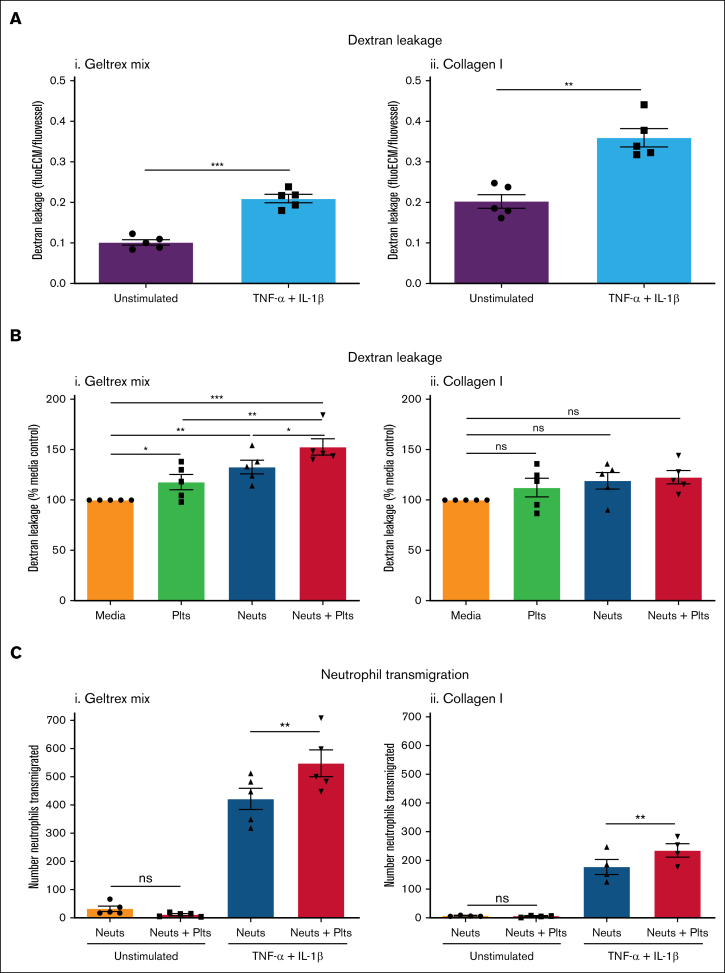
**Platelets promote inflammation in inflamed vessels.** (A) Vessel permeability increases during inflammation. Vessels were cultured against Geltrex mix (i) or collagen I (ii) for 1 week and then stimulated overnight with 10 ng/mL TNF-α + 10 ng/mL IL-1β. FITC-dextran (250 kDa) was added for 45 minutes and then fluorescence imaged on an EVOS M5000. Dextran leakage was calculated by dividing the fluorescence in the ECM channel by the fluorescence in the vessel channel. (B) Platelets and neutrophils enhance permeability in inflamed Geltrex mix vessels. Plts and neuts were isolated from human blood and resuspended in EGM-FCS. They were then perfused through the vessels for 1.5 hours. FITC-dextran (250 kDa) was added as described in panel A. Dextran leakage was normalized to the media only control. (C) Platelets enhance neutrophil transmigration. Vessels were fixed, blocked, and stained with Hoechst-33342 and α-CD31. Z-stacks were taken on a Leica SP5 confocal microscope. Number of neutrophils transmigrated was analyzed using the Cell Counter plug in in FIJI. N = 5 independent experiments/blood donors (N = 4 in panel Cii), n = 2 chips/condition. Mean ± SEM; ∗*P* < .05, ∗∗*P* < .01, ∗∗∗*P* < .001; ns, nonsignificant.

Inflammation increases vascular permeability.^33^ Stimulation of vessels with inflammatory cytokines TNF-α and IL-1β significantly increased FITC-dextran leakage, by approximately twofold (Figure 2A). Collagen I vessels were twice as leaky as Geltrex mix vessels, in both unstimulated and inflamed conditions.

Perfusion of inflamed vessels with platelets and/or neutrophils significantly increased FITC-dextran leakage from Geltrex mix vessels (Figure 2Bi; supplemental Figure 3B). Platelets alone increased FITC-dextran leakage to 117.8% ± 7.5% of control, in marked contrast to their effect in unstimulated vessels. Neutrophils alone increased FITC-dextran leakage to 132.7% ± 6.8% of control. Moreover, when both platelets and neutrophils were perfused, FITC-dextran leakage was increased to 152.6% ± 8.1% of control. Collagen I vessels showed a trend toward increasing FITC-dextran leakage with platelet and/or neutrophil perfusion but this was not statistically significant (Figure 2Bii; supplemental Figure 4B), perhaps because these vessels already exhibited higher FITC-dextran leakage (Figure 2A). Inflammation–induced neutrophil transmigration. Interestingly, in contrast to what we found previously with stimulation by a low concentration (1.6 ng/mL) of TNF-α,^11^ the combined TNF-α and IL-1β stimulus promoted neutrophil transmigration in collagen I vessels in the absence of a chemotactic fMLP (*N*-formyl-methionyl-leucyl-phenylalanine) gradient. This suggests that a threshold of vessel inflammation exists for neutrophils to transmigrate into collagen I, that can be overcome with a stronger stimulus. Despite the strength of the inflammatory signal, the presence of platelets significantly enhanced neutrophil transmigration across inflamed vessels in both Geltrex mix and collagen I vessels (Figure 2C). Together, these results demonstrate a proinflammatory role for platelets during inflammation.

**Figure 3.**
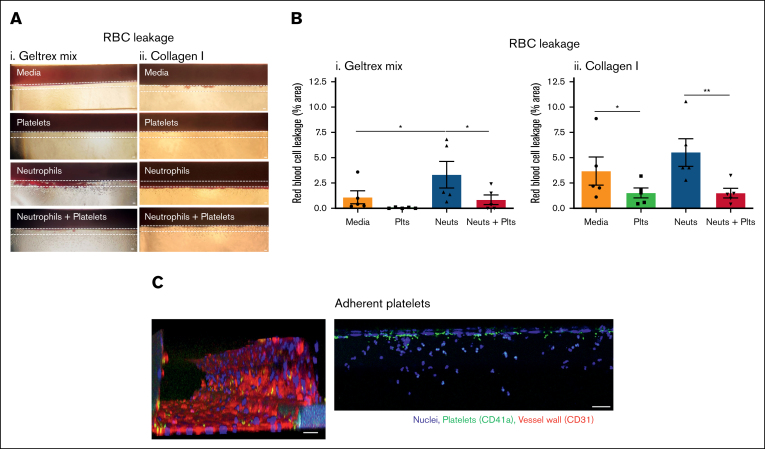
**Platelets prevent RBC leakage in inflamed vessels.** (A) RBC leakage requires neutrophil transmigration in Geltrex mix vessels. Vessels were cultured against Geltrex mix (i) or collagen I (ii) for 1 week, and then stimulated overnight with 10 ng/mL TNF-α + 10 ng/mL IL-1β. Plts and neuts were isolated from human blood and resuspended in EGM-FCS. They were then perfused through the vessels for 1.5 hours. RBCs were added to the vessels for 30 minutes and then RBC leakage was imaged using an iPhone through a bright-field microscope. Dashed lines highlight the phase guides, the top of which marks the vessel wall. (B) Platelets prevent RBC leakage from Geltrex mix and collagen I vessels. RBC leakage was quantified in FIJI. (C) Single platelets adhere to inflamed vessels. Vessels were fixed, blocked, and stained with Hoechst-33342 (blue), α-CD41a (green), and α-CD31 (red). Z-stacks were taken on a Leica SP5 confocal microscope. N = 5 independent experiments per blood donor, n = 2 chips per condition. Mean ± SEM; ∗*P* < .05 and ∗∗*P* < .01. Scale bars are representative of 50 μm.

Next, we investigated the leakage of RBCs during inflammation. Differences in the propensity of vessels to bleed were seen depending on the ECM composition. In inflamed Geltrex mix vessels, very little RBC leakage occurred in the media control or in the presence of platelets alone. When neutrophils were perfused, there was a significant induction of RBC leakage (bleeding) from the vessels (Figure 3Ai-Bi). This was significantly inhibited by coperfusion of platelets. Immunofluorescence imaging revealed presence of adherent platelets within the vessel wall (Figure 3C). The platelets did not form large aggregates but bound singly or in low numbers. Together, this supports data from mouse models that platelets have a dual role in promoting inflammation while simultaneously preserving vessel integrity. Moreover, to the best of our knowledge, this represents the first in vitro model of inflammatory hemostasis.

Interestingly, and in contrast to the Geltrex mix vessels, RBC leakage occurred in inflamed collagen I vessels in the absence of platelets or neutrophils (Figure 3Aii-Bii). This suggests that the level of inflammatory insult or damage required to induce vessel barrier dysfunction can vary depending on the underlying vascular bed. Despite this difference, platelets still significantly inhibited RBC leakage, both in the presence and absence of neutrophils.

### Angiogenic vessel leakiness is reduced by platelets

**Figure 4.**
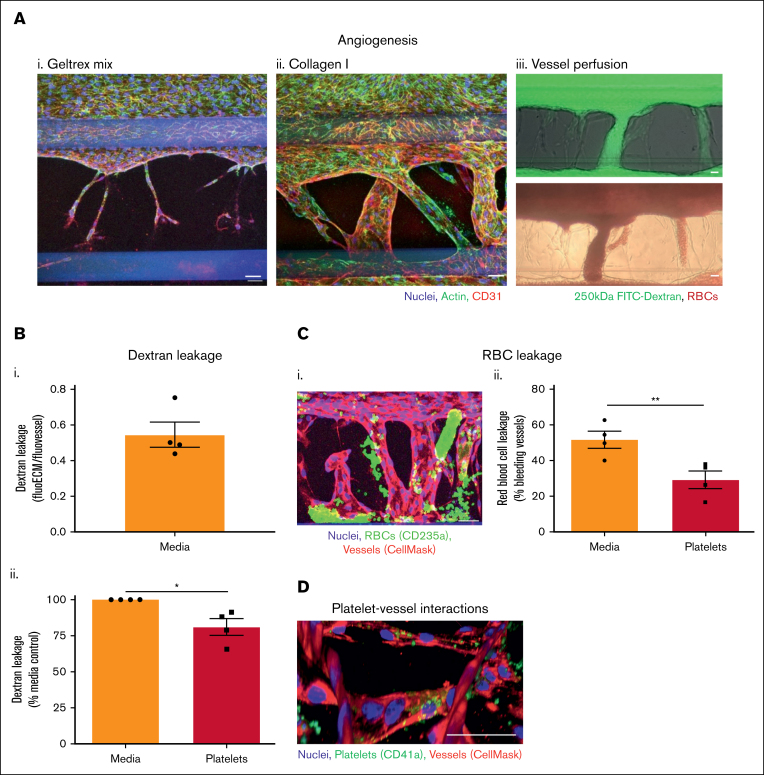
**Platelets preserve integrity of angiogenic vessels.** (A) Perfusable angiogenic vessels can be induced into collagen I ECM. Vessels were cultured against Geltrex mix (i) or collagen I (ii) for 2 days in a 3-lane OrganoPlate before addition of an angiogenesis-inducing cocktail of vascular endothelial growth factor (VEGF), S1P, basic fibroblast growth factor (bFGF), and PMA to the bottom channel for 8 days. Vessels were fixed, blocked, and stained with Hoechst-33342 (blue), phalloidin-FITC (green), and α-CD31 (red). Z-stacks were taken on a Leica SP5 confocal microscope. (iii) After 8 days of angiogenesis induction, collagen I vessels were perfused with 250 kDa FITC-dextran (green) or RBCs for 45 minutes and imaged on an EVOS M5000/bright-field microscope. (B) Platelets reduce angiogenic vessel permeability. Platelets were isolated from human blood and resuspended in EGM-FCS. They were then perfused through the vessels for 1.5 hours 250 kDa FITC-dextran was added to the vessels for the last 45 minutes of platelet perfusion, and then fluorescence imaged on an EVOS M5000. Dextran leakage was calculated by dividing the fluorescence in the ECM by the fluorescence in the vessel. Data were then normalized to the media only control. (C) Platelets prevent RBC leakage from angiogenic vessels. (i) Vessels were prestained with CellMask Deep Red Plasma Membrane stain (red). After the process described in panel B, RBCs were added to the vessels for 30 minutes. Vessels were then fixed, blocked, and stained with Hoechst-33342 (blue), and α-CD235a (green). Z-stacks were taken on a Leica SP5 confocal microscope. (ii) RBC leakage was quantified in FIJI. (D) Platelets bind singly to angiogenic vessels. Vessels (red) were fixed, blocked, and stained with Hoechst-33342 (blue), and α-CD41a (green). Z-stacks were taken on a Leica SP5 confocal microscope. 3D reconstructions were generated in FIJI. N = 4 independent experiments per blood donor, n = 3 chips per condition. Mean ± SEM; ∗*P* < .05 and ∗∗*P* < .01. Scale bars are representative of 50 μm.

Platelets have also previously been found to prevent macromolecule and RBC leakage in vivo during angiogenesis.^29^^,^^30^ To model this in vitro, we used the 3-lane OrganoPlate to induce endothelial sprouting out of the main vessel and into the adjacent matrix, toward a gradient of angiogenesis-inducing factors in the bottom channel. After 8 days, small sprouts were induced into Geltrex mix, but these failed to develop further (Figure 4Ai). In contrast, vessels formed across the entire width of the collagen I ECM, undergoing anastomosis into the bottom channel (Figure 4Aii). The vessels formed in collagen I were perfusable with both FITC-dextran and RBCs (Figure 4Aiii).

Angiogenic neovessels that formed across the collagen I channel were leakier to FITC-dextran than the large vessels formed against Geltrex mix or collagen I in the 2-lane OrganoPlate (Figure 4Bi; compared with Figure 2Ai). Perfusion of platelets through the vessels significantly reduced the dextran leakage, as observed in the unstimulated vessels in the 2-lane plate (Figure 4Bii). When RBCs were added to the angiogenic vessels, bleeding into the collagen I matrix was observed (Figure 4Ci). RBC leakage was defined as CD235a^+^ RBCs outside areas occupied by the vessel cell mask; note that vessels were gently washed before imaging, removing most RBCs inside the perfusable vessels. Overall, 51.8% ± 4.7% of the vessels exhibited RBC leakage. This was reduced to 29.3% ± 4.9% by platelet perfusion (Figure 4Cii). Consistent with our observations of inflamed vessels in the 2-lane plate, platelets were found binding within the vessel walls of the angiogenic vessels, again singly or in low numbers (Figure 4D). Together, these data show that platelets reduce the leak of macromolecules and hemorrhage of RBCs, supporting the barrier function of angiogenic neovessels.

## Discussion

Platelets regulate vascular inflammation and neutrophil infiltration into tissues. Here, we developed multicellular models of inflammation and inflammatory hemostasis that incorporate platelets and RBCs. These models demonstrate how the inclusion of platelets alters vascular integrity in organ-on-a-chip models, creating a more complete representation of how vascular integrity is regulated in vivo*.* To investigate the role of platelets we built on our previous inflammation-on-a-chip model,^11^ on a background of 2 different ECM compositions. We successfully perfused platelets, neutrophils, and RBCs through endothelial vessels and measured the influence of platelets on vascular permeability, neutrophil transmigration, and leakage of RBCs (which we interpret as bleeding). The effect of platelets was context dependent, preventing leakage of macromolecules from unstimulated vessels but promoting it in inflamed vessels. In addition, platelets prevented leakage of RBCs during inflammation, while simultaneously enhancing neutrophil transmigration at these sites, recapitulating in vivo mouse models of inflammatory hemostasis.^17^^,^^23^ Platelets also attenuated leakage of RBCs from angiogenic neovessels. This work demonstrates the potential to study cell-cell and cell-microenvironment interactions in complex 3D in vitro models. It also emphasizes the importance of modeling the role of platelets in inflammation. The commercial availability of the OrganoPlate enables the models to be highly reproducible and transferable to other laboratories, which is particularly important when investigating subtle and intricate cell-cell interactions. Future studies could use these models to investigate the mechanisms by which these cells influence each other, and whether they could be targeted therapeutically. Importantly, our models use human cells, increasing their translational potential compared with murine models.

Platelets reduced the leak of macromolecules from noninflamed vessels. Although HUVEC vessels cultured in the 2-lane OrganoPlate exhibited a high level of FITC-dextran retention, there was some leak of FITC-dextran into the ECM compartment. Vessels cultured against collagen I were leakier than those cultured against Geltrex mix, indicating comparatively lower barrier function. Permeability to FITC-dextran was significantly reduced by just 90 minutes of perfusion with platelets. Very little platelet adherence to the vessel wall was observed, suggesting that the effect could be due to soluble mediators released by platelets,^13^^,^^15^ or transient on/off binding.^34^ Platelets have previously been shown to have a protective effect on the resting endothelium in in vivo^16^ and in vitro models.^35^ Early studies in rabbits found the resting endothelium to become thin, fragile, and leaky after only 6 hours of thrombocytopenia.^36^ Incorporation of platelets into organ-on-a-chip models could therefore be important to represent the in vivo vasculature more accurately.

Platelets increased macromolecule leakage from inflamed vessels in an ECM-dependent manner. Stimulation with TNF-α and IL-1β significantly increased vessel FITC-dextran permeability. In contrast to unstimulated vessels, addition of platelets further enhanced FITC-dextran leak of inflamed Geltrex mix vessels. This enhancement has been observed previously in mouse models^13^, ^14^, ^15^ but, to our knowledge, not in vitro with human cells and platelets. How platelets reduce permeability in unstimulated vessels but promote it during inflammation is not well understood but could be due to the upregulation of different ligands on the endothelial surface during inflammation, which either stimulate platelets to release proinflammatory mediators such as serotonin^15^^,^^37^ or generate downstream signaling within the endothelium leading to junctional changes.^38^ The permeability of inflamed Geltrex mix vessels was also enhanced by neutrophils, as previously established in vivo*.*^39^, ^40^, ^41^

Interestingly, the ECM composition influenced the contribution of platelets and neutrophils. Inflamed collagen I vessels did not exhibit the same degree of enhancement of permeability by platelets and neutrophils as Geltrex mix. This could be because they were already moderately leaky, and so no further structural changes to the endothelial barrier could be induced by platelet- or neutrophil-endothelial cross talk. ECM composition and structure has previously been shown to modulate endothelial phenotype. Studies have found permeability to increase with increasing stiffness.^42^^,^^43^ Because Geltrex is reported to have a lower stiffness than collagen I (900^44^ vs 1200^45^ Pa), this could explain the lower permeability on this ECM. Additionally, the availability of different ligands within Geltrex may modulate the phenotype alongside any mechanotransductive effects.

Although many studies have used 70-kDa dextran to model albumin leakage, other plasma proteins that leak from the vessel during inflammation, such as antibodies, are larger than 70 kDa.^25^ Therefore, by using 250 kDa we could model the leakage of these larger sized proteins, which play important roles at inflammatory sites. This size of dextran has been used in other studies investigating vascular leakage,^46^^,^^47^ including in a vessel-on-a-chip device.^48^ Future studies could use dextrans of different sizes to investigate leakage of different proteins.

Platelets also enhanced neutrophil transmigration, in both Geltrex mix and collagen I inflamed vessels. Many previous in vivo models have found platelets to be important in neutrophil recruitment to inflamed vessels^13^^,^^14^^,^^49^, ^50^, ^51^, ^52^ although this is not always the case.^53^^,^^54^ For example, in a colitis model, depletion of platelets prevented neutrophil recruitment and transmigration, but neutrophil depletion had no effect on platelet recruitment,^55^ although most neutrophils colocalized with adherent platelets, and neutrophils that were recruited by platelets were more activated. Our model could be used to investigate how platelets enhance neutrophil transmigration.

Platelets inhibited RBC leakage (bleeding) from inflamed vessels. Multiple in vivo studies using diverse mouse inflammation models across different organs reported RBC leakage at sites of inflammation during thrombocytopenia.^16^, ^17^, ^18^, ^19^, ^20^, ^21^, ^22^, ^23^, ^24^ Similarly, in our model, RBCs leaked from inflamed vessels when platelets were absent, but this was significantly inhibited when platelets were present. Neutrophil infiltration, rather than just inflammation-induced permeability, was found to be required in several in vivo studies, because depletion of neutrophils and platelets prevented the bleeding^17^^,^^20^^,^^23^^,^^56^^,^^57^ and the presence of activated neutrophils alone without transmigration did not induce bleeding.^17^ This is mimicked by our Geltrex mix vessels, in which inflammation alone induced a small amount of RBC leakage, and this was significantly increased by the addition of neutrophils. In contrast, not all inflammatory bleeding in vivo could be prevented by neutrophil depletion,^58^^,^^59^ suggesting that different vascular beds may be more or less susceptible to damage, and different models of inflammation could induce more or less damage, producing variability in the induction of bleeding by infiltrating neutrophils. Accordingly, bleeding occurred in our inflamed collagen I vessels without neutrophils and was not increased by neutrophils. The difference in bleeding between Geltrex mix and collagen I vessels supports that the underlying vascular bed of the vessel determines the propensity to bleed in response to damaging stimuli. The use of different ECMs in organ-on-a-chip models therefore allows different vascular beds to be modeled and is an important strength of these models.

Our new model of inflammatory hemostasis will be a valuable tool for understanding how platelets perform this role. Platelets were observed binding within the vessel walls but no large thrombi were present, supporting data from mouse models that platelet aggregation is not required to prevent bleeding during inflammation.^19^^,^^23^ However, the relative contribution of different platelet receptors appears to vary between different vascular beds.^21^ Our model allows the variation of individual components, such as ECM composition, as described in this study, which will allow the contribution of different platelet receptors and responses to be compared. A limitation of our current model is that a single inflammatory stimulus is used, although the model could also be triggered by other stimuli such as immune complexes. In principle, tissue-resident cells such as macrophages or mast cells could also be embedded in the ECM, giving more complex patterns of inflammatory stimulation. However, this would further increase the complexity of the model. An inherent limitation of in vitro models is that there is a need to balance model simplicity, which increases intralaboratory and interlaboratory reproducibility, and model complexity, which may increase the extent to which the model accurately mimics the in vivo setting. This balance is shown in our models, in which the addition of platelets increases the complexity of the model but also provides an important factor (and potential repertoire of drug targets) that would normally be present in vivo. The interactions captured would not have been possible in a simple 2D format.

Other vessel-on-a-chip models of bleeding have been developed. In one, a valve is opened under an endothelial layer, creating a “wound” over which blood is then perfused.^60^ RBCs initially leak from the wound before a hemostatic plug is formed by platelets, and bleeding prevented. In another study, a small needle was used to create the vessel injury.^61^ Both studies used endothelial cells and blood components to model mechanical injury of vessels and subsequent hemostasis. In contrast, the injury in our model is inflammatory and on a much smaller scale, with platelet aggregation/coagulation not required to maintain hemostasis. Another model has cultured 3D HUVEC microvessels within collagen I and demonstrated their ability to retain 70-kDa dextran.^62^ They also perfused platelets and found that they interacted with the vessel under inflamed conditions but not to unstimulated vessels. However, they did not study the effect of platelets and/or immune cells on the barrier function of the vessel. We believe that our model bridges these classical hemostasis and inflammation models, to produce a novel model of inflammatory hemostasis. Finally, we investigated whether our approach could be extended to other contexts in which platelets protect vascular integrity. Dysregulated angiogenesis occurs in several diseases including cancer and other chronic inflammatory diseases such as asthma and rheumatoid arthritis.^63^ Similarly to inflamed vessels, these angiogenic vessels are inherently leaky to macromolecules, and sometimes RBCs.^29^^,^^63^^,^^64^ Recent mouse models have found increased plasma and RBC leakage during angiogenesis when platelets were depleted.^18^^,^^20^^,^^56^^,^^65^ We therefore developed an angiogenesis model to investigate whether the protective role of platelets can be recapitulated in vitro.

We moved to the 3-lane OrganoPlate, using the bottom channel of the chip to add angiogenic stimuli that would induce invasion of endothelial cells out of the main vessel and into the ECM. Interestingly, successful neovessel formation was only possible in collagen I and not Geltrex mix. This is in sharp contrast to invasion of the ECM by neutrophils, which we previously found to be hindered in collagen I.^11^ A previous study found that invasion of ECM by epithelial cells was dependent on the presence of collagen fibrils and that cells did not invade Matrigel.^66^ In support of this, another study found that invasion occurs along aligned collagen fibrils.^67^ These findings could explain the lack of angiogenesis into Geltrex mix.

The vessels induced within collagen I formed lumens that were perfusable with fluorescent dextran and RBCs. The vessel-on-a-chip model of angiogenesis was leakier to FITC-dextran than Geltrex mix or collagen I vessels. In this model, and as seen in vivo, no inflammation or neutrophil transmigration was required to induce bleeding, with RBC leakage observed in ∼50% of unstimulated neovessels. It is challenging to directly compare the level of RBC leakage because the angiogenic vessels are variable in shape and size compared with the collagen I and Geltrex mix vessels, which are of standardized dimensions. However, the RBC leakage appeared to be more severe in the angiogenic vessels, often occurring throughout the entire length of the vessel.

Platelets promoted vascular integrity of angiogenic vessels. Both FITC-dextran and RBC leakage were significantly reduced by platelets in the angiogenesis-on-a-chip model. Confocal microscopy revealed single platelets binding within the angiogenic vessel walls. This provides further evidence that platelets can preserve vessel integrity without aggregation or coagulation, even when vessels are severely leaky. Previous studies that developed in vitro angiogenic vessels have perfused dextrans,^68^, ^69^, ^70^ 1- to 7-μm beads,^68^^,^^70^^,^^71^ immune cells,^70^ and RBCs.^72^^,^^73^ However, this is the first study that has perfused isolated platelets, and subsequently analyzed their interactions with the vessel wall and effects on vessel function. In our model, platelets were perfused for 90 minutes after 8 days of angiogenesis, allowing us to investigate the role of platelets on late stage of angiogenesis. However, platelets promote angiogenesis in vivo at early and late stages.^29^ By adding platelets at different points, our model could be used to investigate the role of platelets at different stages.

In summary, our multicellular models demonstrate the influence of platelets on vascular integrity in inflammation and angiogenesis, successfully recapitulating the roles of platelets in vivo. Additionally, our models provide a platform to investigate the mechanisms by which platelets perform these multifaceted and important roles.

Conflict-of-interest disclosure: K.J. and K.M.H. are employees of AstraZeneca. The remaining authors declare not competing financial interests.

## References

[1] Mayadas TN, Cullere X, Lowell CA (2014). The multifaceted functions of neutrophils. Annu Rev Pathol.

[2] Gierlikowska B, Stachura A, Gierlikowski W, Demkow U (2021). Phagocytosis, degranulation and extracellular traps release by neutrophils—the current knowledge, pharmacological modulation and future prospects. Front Pharmacol.

[3] Chen L, Deng H, Cui H (2018). Inflammatory responses and inflammation-associated diseases in organs. Oncotarget.

[4] Schett G, Neurath MF (2018). Resolution of chronic inflammatory disease: universal and tissue-specific concepts. Nat Commun.

[5] Mestas J, Hughes CCW (2004). Of mice and not men: differences between mouse and human immunology. J Immunol.

[6] Zschaler J, Schlorke D, Arnhold J (2014). Differences in innate immune response between man and mouse. Crit Rev Immunol.

[7] Jensen C, Teng Y (2020). Is it time to start transitioning from 2D to 3D cell culture?. Front Mol Biosci.

[8] Low LA, Mummery C, Berridge BR, Austin CP (2020). Tagle DA Organs-on-chips: into the next decade. Nat Rev Drug Discov.

[9] Ma C, Peng Y, Li H, Chen W (2021). Organ-on-a-chip: a new paradigm for drug development. Trends Pharmacol Sci.

[10] Huh D, Matthews BD, Mammoto A, Montoya-Zavala M, Yuan Hsin H, Ingber DE (2010). Reconstituting organ-level lung functions on a chip. Science.

[11] Riddle RB, Jennbacken K, Hansson KM, Harper MT (2022). Endothelial inflammation and neutrophil transmigration are modulated by extracellular matrix composition in an inflammation-on-a-chip model. Sci Rep.

[12] Manne BK, Xiang SC, Rondina MT (2017). Platelet secretion in inflammatory and infectious diseases. Platelets.

[13] Hara T, Shimizu K, Ogawa F (2010). Platelets control leukocyte recruitment in a murine model of cutaneous arthus reaction. Am J Pathol.

[14] Asaduzzaman M, Lavasani S, Rahman M (2009). Platelets support pulmonary recruitment of neutrophils in abdominal sepsis. Crit Care Med.

[15] Cloutier N, Paré A, Farndale RW (2012). Platelets can enhance vascular permeability. Blood.

[16] Gupta S, Konradt C, Corken A (2020). Hemostasis vs. homeostasis: platelets are essential for preserving vascular barrier function in the absence of injury or inflammation. Proc Natl Acad Sci U S A.

[17] Hillgruber C, Pöppelmann B, Weishaupt C (2015). Blocking neutrophil diapedesis prevents hemorrhage during thrombocytopenia. J Exp Med.

[18] Ho-Tin-Noé B, Goerge T, Cifuni SM, Duerschmied D, Wagner DD (2008). Platelet granule secretion continuously prevents intratumor hemorrhage. Cancer Res.

[19] Boulaftali Y, Hess PR, Getz TM (2013). Platelet ITAM signaling is critical for vascular integrity in inflammation. J Clin Invest.

[20] Ho-Tin-Noé B, Carbo C, Demers M, Cifuni SM, Goerge T, Wagner DD (2009). Innate immune cells induce hemorrhage in tumors during thrombocytopenia. Am J Pathol.

[21] Rayes J, Jadoui S, Lax S (2018). The contribution of platelet glycoprotein receptors to inflammatory bleeding prevention is stimulus and organ dependent. Haematologica.

[22] Goerge T, Ho-Tin-Noe B, Carbo C (2008). Inflammation induces hemorrhage in thrombocytopenia. Blood.

[23] Gros A, Syvannarath V, Lamrani L (2015). Single platelets seal neutrophil-induced vascular breaches via GPVI during immune-complex-mediated inflammation in mice. Blood.

[24] Deppermann C, Kraft P, Volz J (2017). Platelet secretion is crucial to prevent bleeding in the ischemic brain but not in the inflamed skin or lung in mice. Blood.

[25] Egawa G, Nakamizo S, Natsuaki Y, Doi H, Miyachi Y, Kabashima K (2013). Intravital analysis of vascular permeability in mice using two-photon microscopy. Sci Rep.

[26] Loria GD, Romagnoli PA, Moseley NB, Rucavado A, Altman JD (2013). Platelets support a protective immune response to LCMV by preventing splenic necrosis. Blood.

[27] Carbo C, del Conde I, Duerschmied D (2009). Petechial bleeding after sunburn in a patient with mild thrombocytopenia. Am J Hematol.

[28] Sangwan A, Tewari S, Narula SC, Sharma RK, Sangwan P (2013). Significance of periodontal health in primary immune thrombocytopenia- a case report and review of literature. J Dent.

[29] Kisucka J, Butterfield CE, Duda DG (2006). Platelets and platelet adhesion support angiogenesis while preventing excessive hemorrhage. Proc Natl Acad Sci USA.

[30] Li R, Ren M, Chen N (2014). Presence of intratumoral platelets is associated with tumor vessel structure and metastasis. BMC Cancer.

[31] Wei H, Malcor JDM, Harper MT (2018). Lipid rafts are essential for release of phosphatidylserine-exposing extracellular vesicles from platelets. Sci Rep.

[32] Schindelin J, Arganda-Carreras I, Frise E (2012). Fiji: an open-source platform for biological-image analysis. Nat Methods.

[33] Kim MH, Curry FRE, Simon SI (2009). Dynamics of neutrophil extravasation and vascular permeability are uncoupled during aseptic cutaneous wounding. Am J Physiol Cell Physiol.

[34] Jenne CN, Wong CHY, Petri B, Kubes P (2011). The use of spinning-disk confocal microscopy for the intravital analysis of platelet dynamics in response to systemic and local inflammation. PLoS One.

[35] Lo SK, Burhop KE, Kaplan JE, Malik AB (1988). Role of platelets in maintenance of pulmonary vascular permeability to protein. Am J Physiol.

[36] Kitchens C, Weiss L (1975). Ultrastructural changes of endothelium associated with thrombocytopenia. Blood.

[37] Retamal JS, Grace MS, Dill LK (2021). Serotonin-induced vascular permeability is mediated by transient receptor potential vanilloid 4 in the airways and upper gastrointestinal tract of mice. Lab Invest.

[38] Kong J, Yao C, Dong S (2021). ICAM-1 activates platelets and promotes endothelial permeability through VE-cadherin after insufficient radiofrequency ablation. Adv Sci.

[39] Finsterbusch M, Voisin MB, Beyrau M, Williams TJ, Nourshargh S (2014). Neutrophils recruited by chemoattractants in vivo induce microvascular plasma protein leakage through secretion of TNF. J Exp Med.

[40] DiStasi MR, Ley K (2009). Opening the flood-gates: how neutrophil-endothelial interactions regulate permeability. Trends Immunol.

[41] Kenne E, Rasmuson J, Renné T (2019). Neutrophils engage the kallikrein-kinin system to open up the endothelial barrier in acute inflammation. FASEB J.

[42] Huynh J, Nishimura N, Rana K (2011). Age-related intimal stiffening enhances endothelial permeability and leukocyte transmigration. Sci Transl Med.

[43] Pérez-Rodríguez S, Huang SA, Borau C, García-Aznar JM, Polacheck WJ (2021). Microfluidic model of monocyte extravasation reveals the role of hemodynamics and subendothelial matrix mechanics in regulating endothelial integrity. Biomicrofluidics.

[44] Kothapalli C, Mahajan G, Farrell K (2020). Substrate stiffness induced mechanotransduction regulates temporal evolution of human fetal neural progenitor cell phenotype, differentiation, and biomechanics. Biomater Sci.

[45] Paszek MJ, Zahir N, Johnson KR (2005). Tensional homeostasis and the malignant phenotype. Cancer Cell.

[46] Gao S, Wake H, Gao Y (2019). Histidine-rich glycoprotein ameliorates endothelial barrier dysfunction through regulation of NF-κB and MAPK signal pathway. Br J Pharmacol.

[47] Maeso-Alonso L, Alonso-Olivares H, Martínez-García N (2022). p73 is required for vessel integrity controlling endothelial junctional dynamics through Angiomotin. Cell Mol Life Sci.

[48] Barbato MG, Pereira RC, Mollica H, Palange AL, Ferreira M, Decuzzi P (2021). A permeable on-chip microvasculature for assessing the transport of macromolecules and polymeric nanoconstructs. J Colloid Interface Sci.

[49] Tamagawa-Mineoka R, Katoh N, Ueda E, Takenaka H, Kita M, Kishimoto S (2007). The role of platelets in leukocyte recruitment in chronic contact hypersensitivity induced by repeated elicitation. Am J Pathol.

[50] Zarbock A, Singbartl K, Ley K (2006). Complete reversal of acid-induced acute lung injury by blocking of platelet-neutrophil aggregation. J Clin Invest.

[51] Kuligowski MP, Kitching AR, Hickey MJ (2006). Leukocyte recruitment to the inflamed glomerulus: a critical role for platelet-derived P-selectin in the absence of rolling. J Immunol.

[52] Slaba I, Wang J, Kolaczkowska E, Mcdonald B, Lee WY, Kubes P (2015). Imaging the dynamic platelet-neutrophil response in sterile liver injury and repair in mice. Hepatology.

[53] Hidalgo A, Chang J, Jang JE, Peired AJ, Chiang EY, Frenette PS (2009). Heterotypic interactions enabled by polarized neutrophil microdomains mediate thromboinflammatory injury. Nat Med.

[54] Senaldi G, Piguet PF (1997). Platelets play a role in the pathogenesis of the irritant reaction in mice. J Invest Dermatol.

[55] Vowinkel T, Wood KC, Stokes KY (2007). Mechanisms of platelet and leukocyte recruitment in experimental colitis. Am J Physiol Gastrointest Liver Physiol.

[56] Volz J, Mammadova-Bach E, Gil-Pulido J (2019). Inhibition of platelet GPVI induces intratumor hemorrhage and increases efficacy of chemotherapy in mice. Blood.

[57] Wéra O, Lecut C, Servais L (2020). P2X1 ion channel deficiency causes massive bleeding in inflamed intestine and increases thrombosis. J Thromb Haemost.

[58] Bain W, Olonisakin T, Yu M (2019). Platelets inhibit apoptotic lung epithelial cell death and protect mice against infection-induced lung injury. Blood Adv.

[59] Claushuis TAM, de Vos AF, Nieswandt B (2018). Platelet glycoprotein VI aids in local immunity during pneumonia-derived sepsis caused by gram-negative bacteria. Blood.

[60] Sakurai Y, Hardy ET, Ahn B (2018). A microengineered vascularized bleeding model that integrates the principal components of hemostasis. Nat Commun.

[61] Poventud-Fuentes I, Kwon KW, Seo J (2021). A human vascular injury-on-a-chip model of hemostasis. Small.

[62] Zheng Y, Chen J, Craven M (2012). In vitro microvessels for the study of angiogenesis and thrombosis. Proc Natl Acad Sci U S A.

[63] Yoo SY, Kwon SM (2013). Angiogenesis and its therapeutic opportunities. Mediators Inflamm.

[64] Johnstone C, Rich SE (2018). Bleeding in cancer patients and its treatment: a review. Ann Palliat Med.

[65] Demers M, Ho-Tin-Noé B, Schatzberg D, Yang JJ, Wagner DD (2011). Increased efficacy of breast cancer chemotherapy in thrombocytopenic mice. Cancer Res.

[66] Nguyen-Ngoc KV, Ewald AJ (2013). Mammary ductal elongation and myoepithelial migration are regulated by the composition of the extracellular matrix. J Microsc.

[67] Provenzano PP, Eliceiri KW, Campbell JM, Inman DR, White JG, Keely PJ (2006). Collagen reorganization at the tumor-stromal interface facilitates local invasion. BMC Med.

[68] Kim S, Lee H, Chung M, Jeon NL (2013). Engineering of functional, perfusable 3D microvascular networks on a chip. Lab Chip.

[69] Nashimoto Y, Teraoka Y, Banan Sadeghian R (2018). Perfusable vascular network with a tissue model in a microfluidic device. J Vis Exp.

[70] Paek J, Park SE, Lu Q (2019). Microphysiological engineering of self-assembled and perfusable microvascular beds for the production of vascularized three-dimensional human microtissues. ACS Nano.

[71] Nguyen DHT, Stapleton SC, Yang MT (2013). Biomimetic model to reconstitute angiogenic sprouting morphogenesis in vitro. Proc Natl Acad Sci U S A.

[72] Seo J, Conegliano D, Farrell M (2017). A microengineered model of RBC transfusion-induced pulmonary vascular injury. Sci Rep.

[73] Yeon JH, Ryu HR, Chung M, Hu QP, Jeon NL (2012). In vitro formation and characterization of a perfusable three-dimensional tubular capillary network in microfluidic devices. Lab Chip.

