# Supplementary material for: A multicellular vessel-on-a-chip model reveals context-dependent roles for platelets in inflammation and inflammatory hemostasis
Source: Blood Vessel Thromb Hemost. 2024 Apr 30;1(2):100007. doi: 10.1016/j.bvth.2024.100007 (PMC12320429; doi:10.1016/j.bvth.2024.100007)
Supplement: Supplemental Figures [file BVTH_VTH-2023-000130-mmc1.pdf]

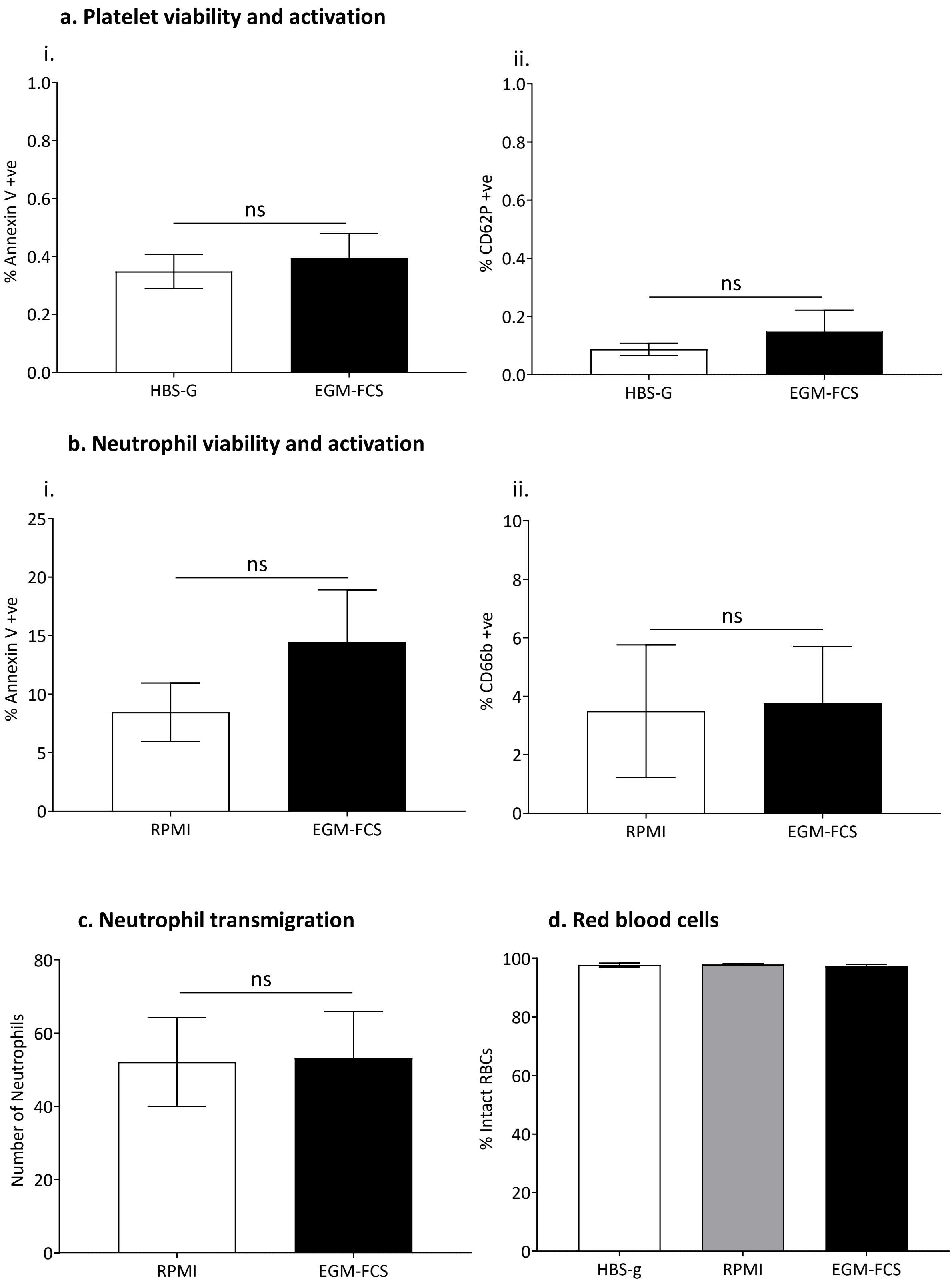

a. Platelet viability and activation
i.
% Annexin V +ve
1.0
0.8
0.6
0.4
0.2
0.0
ns
HBS-G
EGM-FCS
ii.
% CD62P +ve
1.0
0.8
0.6
0.4
0.2
0.0
ns
HBS-G
EGM-FCS
b. Neutrophil viability and activation
i.
% Annexin V +ve
25
20
15
10
5
0
ns
RPMI
EGM-FCS
ii.
% CD66b +ve
10
8
6
4
2
0
ns
RPMI
EGM-FCS
c. Neutrophil transmigration
Number of Neutrophils
80
60
40
20
0
ns
RPMI
EGM-FCS
d. Red blood cells
% Intact RBCs
100
80
60
40
20
0
HBS-g
RPMI
EGM-FCS

Figure S1. Platelets and neutrophils are viable in endothelial medium, EGM, with 10% FCS

a. **Platelet viability is comparable in EGM-FCS vs their typical buffer, HBS-glucose (HBS-g).** Washed platelets were freshly isolated from human blood and resuspended in different media for 1.5 hours, then stained for Annexin V to measure viability (i), or CD62P to measure activation (ii).
b. **Neutrophil viability is comparable in EGM-FCS vs their typical buffer, RPMI.** Neutrophils were freshly isolated from human blood and resuspended in different media for 1.5 hours, then stained for Annexin V to measure viability (i), or CD66b to measure activation (ii).
c. **Neutrophil transmigration is comparable in EGM-FCS vs their typical buffer, RPMI.** Confluent HUVEC monolayers were stimulated with 1.6ng/mL TNF-α for 4 hours. Neutrophils were freshly isolated from human blood, resuspended in RPMI or EGM-FCS, and then added to HUVEC for 1.5 hours. HUVEC were then fixed, blocked, stained with α-CD31 and Hoechst-33342, and imaged on a Leica SP5 confocal microscope. Number of neutrophils per 3 fields of view was quantified using the Cell Counter plug in on FIJI.
d. **RBCs remain intact and unlysed in EGM-FCS, HBS-glucose, and RPMI.** RBCs were freshly isolated from human whole blood and then diluted 1 in 10 in HBS-glucose, EGM, or RPMI. RBCs were incubated for 45 minutes before analysing on a BD Accuri C6 flow cytometer. Intact RBCs were identified based on forward and side scatter profiles. All statistical comparisons were non-significant.

N=5 independent experiments/blood donors. Mean +/- SEM, ns = non-significant.

a. 2-lane OrganoPlate dextran leakage quantification

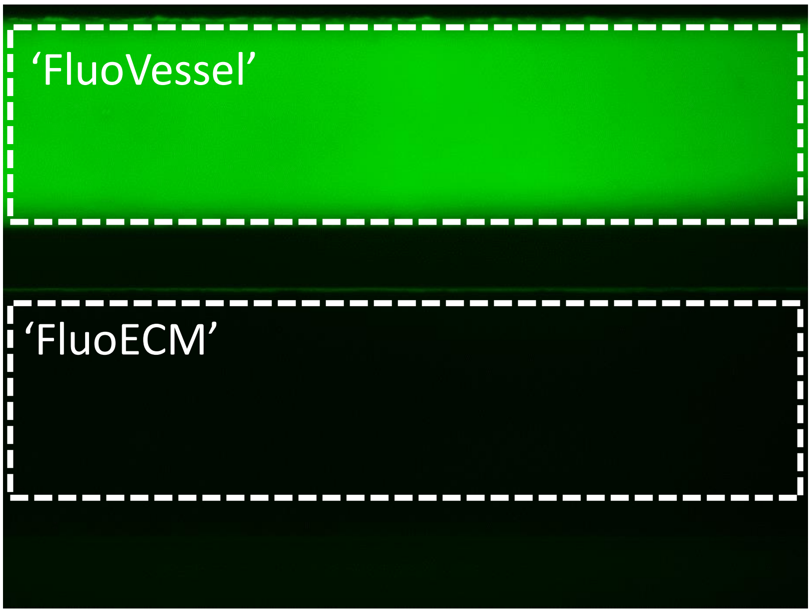

b. 2-lane OrganoPlate RBC leakage quantification

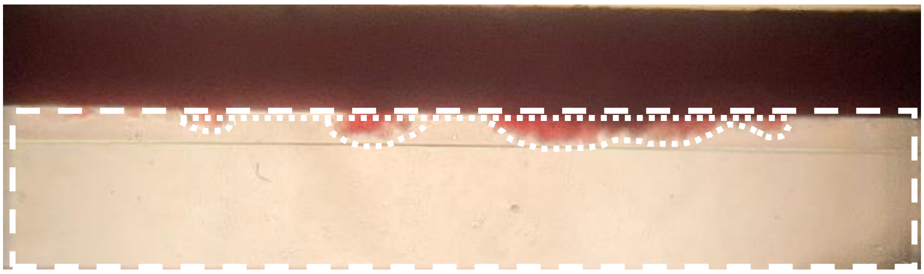

c. 3-lane OrganoPlate angiogenic dextran leakage quantification

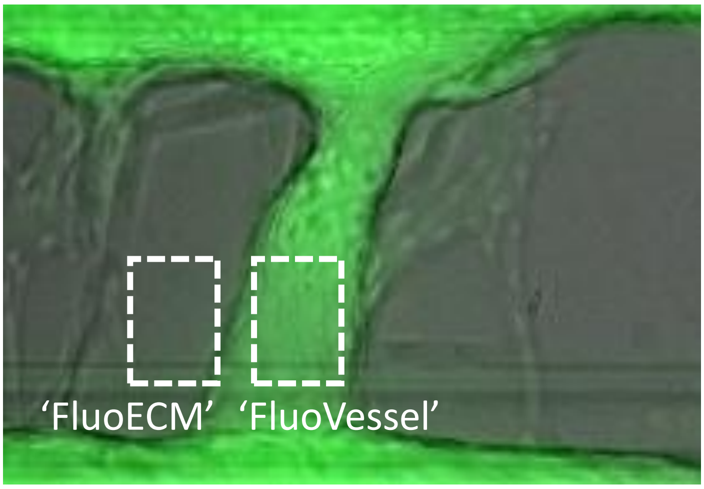

Figure S2. Quantification of dextran and red blood cell leakage.

a. **Quantification of dextran leakage from vessels in the 2-lane OrganoPlate.** A rectangular region of interest (ROI) is drawn around the dextran in the vessel channel, and fluorescence intensity measured ('FluoVessel'). An ROI of identical dimensions is then drawn in the ECM channel, directly below the phaseguide region, and fluorescence intensity measured ('FluoECM'). A ratio of FluoECM/FluoVessel is then calculated.
b. **Quantification of red blood cell (RBC) leakage in the 2-lane OrganoPlate.** A rectangular ROI is drawn around the ECM channel, and area measured. The area of RBC leakage is then outlined and area measured. % Area of RBC leakage is then quantified by dividing area RBC leakage/area of ECM channel, and multiplying by 100.
c. **Quantification of dextran leakage from angiogenic neovessels in the 3-lane OrganoPlate.** A rectangular region of interest (ROI) is drawn within the angiogenic vessel, and fluorescence intensity measured ('FluoVessel'). An ROI of identical dimensions is then drawn in the ECM directly adjacent, and fluorescence intensity measured ('FluoECM'). A ratio of FluoECM/FluoVessel is then calculated.

a. Unstimulated geltrex mix vessels

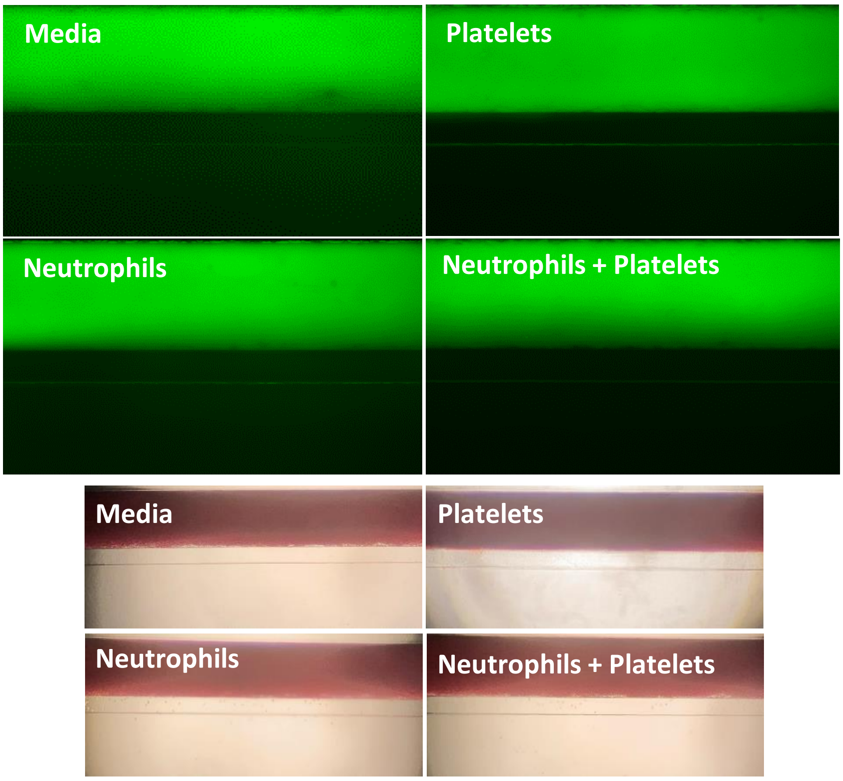

b. TNF-α + IL-1β-Stimulated geltrex mix vessels

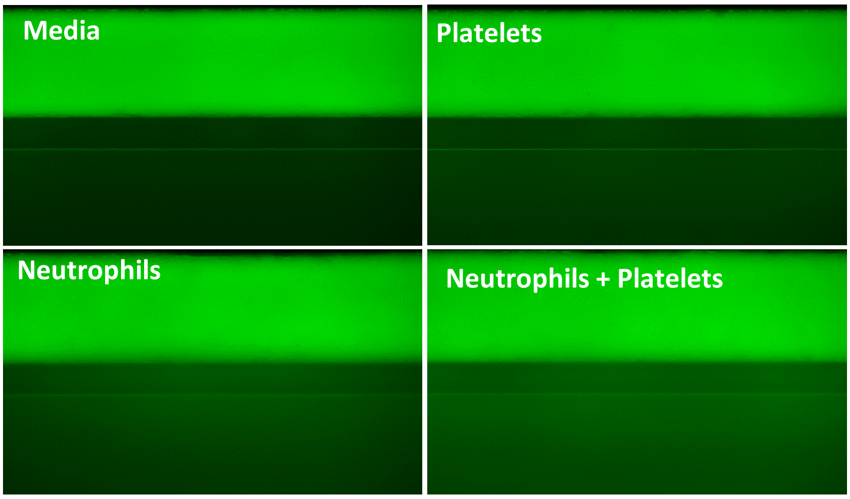

c. Cell-free geltrex mix chip

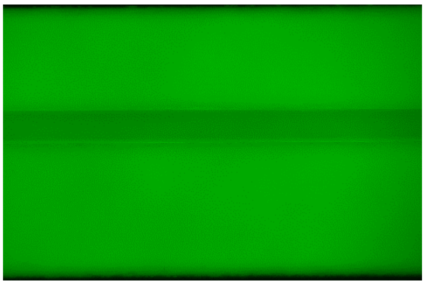

Figure S3. Representative images of dextran leakage from unstimulated and inflamed geltrex mix vessels.

a. **Dextran leakage from unstimulated geltrex mix vessels.** Vessels were cultured against geltrex mix for 1 week. Platelets and neutrophils were isolated from human blood and resuspended in EGM-FCS. They were then perfused through the vessels for 1.5 hours. 250kDa FITC-dextran was added for the last 45 minutes and then fluorescence imaged on an EVOS M5000.
b. **Dextran leakage from TNF-α + IL-1β- stimulated geltrex mix vessels.** Vessels were cultured against geltrex mix for 1 week and then stimulated overnight with 10ng/mL TNF-α + 10ng/mL IL-1β. Platelets and neutrophils were isolated from human blood and resuspended in EGM-FCS. They were then perfused through the vessels for 1.5 hours. 250kDa FITC-dextran was added for the last 45 minutes and then fluorescence imaged on an EVOS M5000.
c. **Dextran leakage in a cell-free chip.** Geltrex mix was added to the ECM channel of a 2-lane OrganoPlate. Instead of seeding HUVEC, only media was added to the top channel, to prevent the ECM from drying out. After 1 week on the rocker, 250kDa FITC-dextran was added for 45 minutes and then fluorescence imaged on an EVOS M5000.

a. Unstimulated collagen I vessels

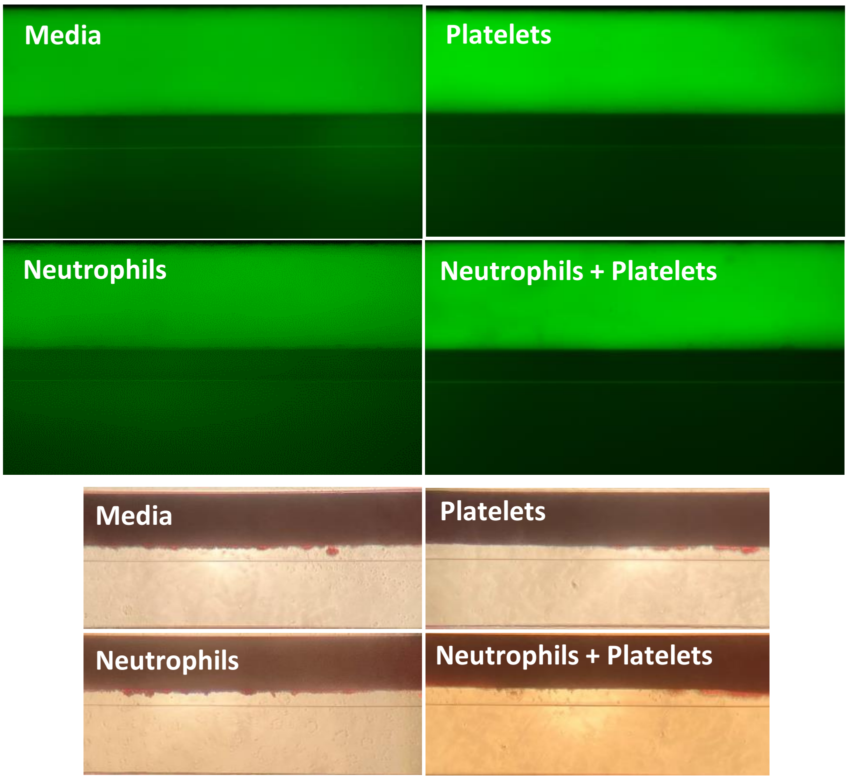

b. TNF-α + IL-1β-Stimulated collagen I vessels

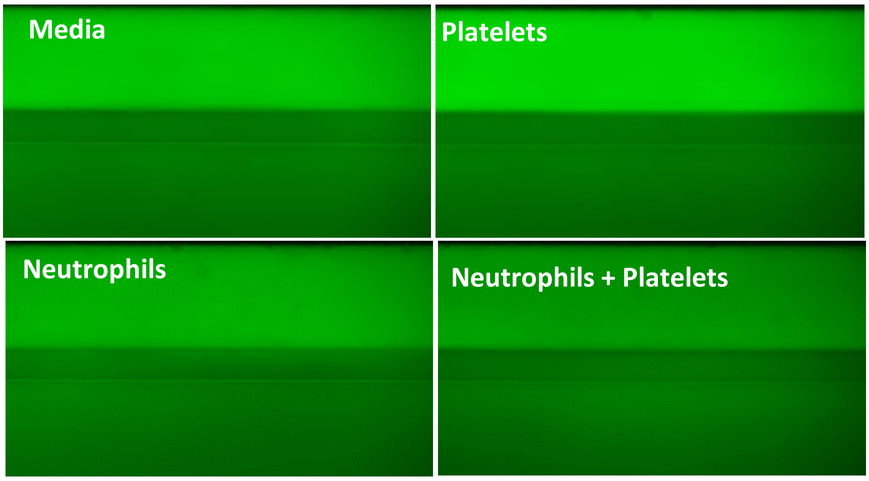

c. Cell-free collagen I chip

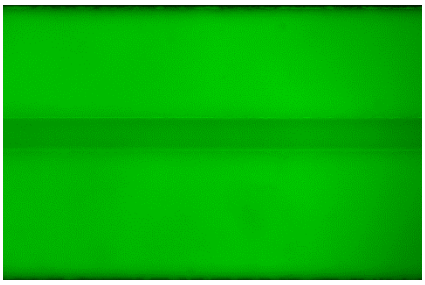

Figure S4. Representative images of dextran leakage from unstimulated and inflamed collagen I vessels.

a. **Dextran leakage from unstimulated collagen I vessels.** Vessels were cultured against collagen I for 1 week. Platelets and neutrophils were isolated from human blood and resuspended in EGM-FCS. They were then perfused through the vessels for 1.5 hours. 250kDa FITC-dextran was added for the last 45 minutes and then fluorescence imaged on an EVOS M5000.
b. **Dextran leakage from TNF-α + IL-1β- stimulated collagen I vessels.** Vessels were cultured against collagen I for 1 week and then stimulated overnight with 10ng/mL TNF-α + 10ng/mL IL-1β. Platelets and neutrophils were isolated from human blood and resuspended in EGM-FCS. They were then perfused through the vessels for 1.5 hours. 250kDa FITC-dextran was added for the last 45 minutes and then fluorescence imaged on an EVOS M5000.
c. **Dextran leakage in a cell-free chip.** Collagen I was added to the ECM channel of a 2-lane OrganoPlate. Instead of seeding HUVEC, only media was added to the top channel, to prevent the ECM from drying out. After 1 week on the rocker, 250kDa FITC-dextran was added for 45 minutes and then fluorescence imaged on an EVOS M5000.
